# The impact of the image conversion factor and image centration on retinal vessel geometric characteristics

**DOI:** 10.3389/fmed.2023.1112652

**Published:** 2023-03-17

**Authors:** Carolin Schanner, Nina Hautala, Franziska G. Rauscher, Aura Falck

**Affiliations:** ^1^Department of Ophthalmology and Medical Research Center, Oulu University Hospital, Oulu, Finland; ^2^PEDEGO Research Unit, University of Oulu, Oulu, Finland; ^3^Institute for Medical Informatics, Statistics, and Epidemiology, Leipzig University, Leipzig, Germany

**Keywords:** retina, retinal microvasculature, retinal image analysis, retinal vessel geometric characteristics (RVGC), ophthalmology, diabetic retinopathy, retinal vessel parameter

## Abstract

**Background:**

This study aims to use fundus image material from a long-term retinopathy follow-up study to identify problems created by changing imaging modalities or imaging settings (e.g., image centering, resolution, viewing angle, illumination wavelength). Investigating the relationship of image conversion factor and imaging centering on retinal vessel geometric characteristics (RVGC), offers solutions for longitudinal retinal vessel analysis for data obtained in clinical routine.

**Methods:**

Retinal vessel geometric characteristics were analyzed in scanned fundus photographs with Singapore-I-Vessel-Assessment using a constant image conversion factor (ICF) and an individual ICF, applying them to macula centered (MC) and optic disk centered (ODC) images. The ICF is used to convert pixel measurements into μm for vessel diameter measurements and to establish the size of the measuring zone. Calculating a constant ICF, the width of all analyzed optic disks is included, and it is used for all images of a cohort. An individual ICF, in turn, uses the optic disk diameter of the eye analyzed. To investigate agreement, Bland-Altman mean difference was calculated between ODC images analyzed with individual and constant ICF and between MC and ODC images.

**Results:**

With constant ICF (*n* = 104 eyes of 52 patients) the mean central retinal equivalent was 160.9 ± 17.08 μm for arteries (CRAE) and 208.7 ± 14.7.4 μm for veins (CRVE). The individual ICFs resulted in a mean CRAE of 163.3 ± 15.6 μm and a mean CRVE of 219.0 ± 22.3 μm. On Bland–Altman analysis, the individual ICF RVGC are more positive, resulting in a positive mean difference for most investigated parameters. Arteriovenous ratio (*p* = 0.86), simple tortuosity (*p* = 0.08), and fractal dimension (*p* = 0.80) agreed well between MC and ODC images, while the vessel diameters were significantly smaller in MC images (*p* < 0.002).

**Conclusion:**

Scanned images can be analyzed using vessel assessment software. Investigations of individual ICF versus constant ICF point out the asset of utilizing an individual ICF. Image settings (ODC vs. MC) were shown to have good agreement.

## 1. Introduction

The fundus of the eye offers a unique opportunity to observe the microvasculature of the human body non-invasively. The analysis of the retinal vessel tree has advanced from measuring vessel diameters (1, 2) around the optic disk to analyzing the geometry of the retinal vascular structure in form of tortuosity measurements (3), branching angles (4), or fractal dimensions (5, 6). According to cross-sectional studies, retinal vascular geometric characteristics (RVGC) (7) can be used as quantifiable and objective early diagnostics in microvascular diseases, e.g., diabetes mellitus (8, 9), hypertension (10, 11), or cardiovascular disorders (12, 13). A few long-term studies also show promising results using RVGC as risk factors for the development of microvascular complications (14, 15). Such complications threaten the quality of life and are a burden to the healthcare system. Identifying patients with a particularly high risk could help allocating resources and support effectively, with the goal to prevent or slow down complications of e.g., diabetes.

Patients with diabetes are recommended to be regularly screened (16), and since the early 2000s fundus photographs started being digitally archived. Under the umbrella of the digital patient archive, caregivers and patients should be able to easily access data, including imaging material in the future.

To facilitate clinical usage of RVGC, reliable reference values need to be available. Establishing reference values is challenging due to a range of factors (17): First of all, measuring a three-dimensional object, as in the fundus of the eye, from a 2D image can only be an estimate. Caliber or angle measurements can therefore always only be a proxy. Thus unsurprisingly, RVGC differ between different vessel assessment software (18, 19). Secondly, even when the same analysis software is used, RVGC can vary for different cameras (due to resolution or field of view) (20) and, third, ocular magnification needs to be factored in to account for differences in axial length and refraction between eyes.

Historically, to measure lesions and compare their sizes between examinations, photographic magnification is corrected for by methods proposed by Littman (21, 22) and Bengtsson and Krakau (23) for the measurement of structures at the background of the eye or for the correction of optic disk measurements on fundus photographs. Those correction factors are device-specific and require additional measurements, such as axial length, and thus can be impractical in clinical practice. Correction factors should be applied with care (24, 25).

Vessel assessment software either processes all analyses in pixels [e.g., VAMPIRE (26, 27)], or employs an average image conversion factor (ICF) computed in a subset of images (e.g., SIVA) (28). This ICF is commonly calculated by the ratio of a reference optic disk (OD) size (e.g., 1,800 μm (29, 30) or 1,850 μm (31, 32), derived by Littmann’s method, based on the assumption of this being the average adult optic disk size) and the average optic disk size of a cohort. In studies where comparisons between different software packages have been attempted, the same conversion method should be used in both cases (19, 28).

To allow comparison of different software or analysis methods to establish RVGC reference values (17, 19), a large public data set is needed, containing fundus images and medical characteristics. This would help determine if associations with systemic variables might be software-dependent. None the less, large cross-sectional studies have shown that associations of RVGC measurements with medical characteristics persist despite a difference in software (18, 19). However, an average ICF does not enable long term follow-up of individual patients, when camera settings change or individual refraction differs between measurements. For example, in a retinopathy screening setting monitoring the development of diabetic retinopathy (DR) is long term (decades) and cameras are updated regularly during that time. However, for a patient in any screening setting it would be important to register RVGC changes between visits and make conclusion based on such developments. An individual ICF could be more reliable considering longitudinal follow-up, as it is based on the OD size of the analyzed eye. In contrast to constant ICF with calculations based on an average OD size, the individual ICF takes into consideration that OD size varies between individuals. We postulate that this could potentially enable comparison of images with variable imaging settings and subsequently changed resolution or field of view and this would also apply to changes in refraction. The use of different camera settings needing adaptation of the average ICF hinders the establishment of reference values from past long-term data (7, 33, 34). There is a need for the acquisition of reference values that are applicable for an individual risk assessment, however, all aforementioned problems and limitations complicate this, limiting the potential for patient care.

This study aims to use fundus image material from a long-term retinopathy follow-up study to identify problems created by changing imaging modalities or settings (e.g., image centering, resolution, viewing angle, illumination wavelength) and to offer solutions for longitudinal retinal vessel analysis. Special emphasis lies on the use of an individual ICF compared to a constant ICF and how the different analysis methods affect associations with obtained medical characteristics.

## 2. Materials and methods

### 2.1. Study population

In the Northern Ostrobothnia Hospital District, Finland, regular diabetes screening has taken place for 30 years. After the duration of 5 years of type 1 diabetes (T1D) or at the latest from the age of 10 years onward, patients are screened annually for diabetic retinopathy (DR) using fundus photography. All 216 children with T1D screened during the years 1989 to 1990 formed the basis for patient selection for the current manuscript (35). The same patients have been investigated from baseline to their 18 years follow-up. The knowledge derived from this study will help facilitate the investigation of the follow-up image data. From the baseline data, all subjects (*N* = 136) in pubertal age (10–15 years) were selected and of those only subjects with optic disk centered images available (*N* = 69) were included in the current study. The ocular fundus of a 10–15-year-old is quite similar in size and other aspects compared to an adult. The growth of the eye is largely completed by then. The rapid growth of the eyeball takes place during the first 1,5 years of life, and an average 3 mm growth between age 1,5 years and adulthood largely happens during childhood years before adolescence (36, 37). Medical data included age, sex, signs of retinopathy graded by the ophthalmologist, stage of puberty, blood pressure, and blood hemoglobin (Hb), serum cholesterol, serum-creatinine, and glycated hemoglobin (HbA1) levels at the time of fundus photography. Glycated hemoglobin was established as HbA1 at the time of its measurement. The state of DR was determined by an ophthalmologist according to the five-scale classification of the Finnish Current care guidelines for DR (38) (Table 1). The Finnish current care guideline is using both the classifications described in Wilkinson et al. (39) and Davis et al. (40) to be used for different purposes. The later more complex Early-Treatment-in-Diabetic-Retinopathy-Study classification has more subgroups and is used more often in studies where there is a need for detailed classification. The five-scale classification [Table 1 of (41)] was adapted from Wilkinson et al. (37).

**TABLE 1:** The classification of retinopathy used for the original fundus images according to the Finnish National Guidelines for Diabetic Retinopathy (36) adapted from Wilkinson et al. (37).

Classification	Definition
No retinopathy	Normal fundus
Mild background retinopathy	Microaneurysms only
Moderate background retinopathy	Microaneurysms, intraretinal hemorrhages, lipid deposits and oedema, microinfarctions, and venous beading, but less than in severe background retinopathy
Severe background retinopathy (preproliferative retinopathy)	Microaneurysms, intraretinal hemorrhages, lipid deposits and oedema, intraretinal microvascular abnormalities (IRMA), microinfarctions and venous beading, and no signs of proliferative retinopathy
Proliferative retinopathy	Neovascularization or resulting vitreous or preretinal hemorrhages, fibrovascular growth or traction retinal detachment

The patients are classified according to the eye with the more advanced retinopathy.

The study has been carried out following good research ethics practices in accordance with the Helsinki Declaration of the World Medical Association. The study was approved by the ethics committee of Oulu University Hospital. All underage patients provided informed assent during the study that resulted in the material used here. Written informed consent was obtained during a reexamination in 2007 from the then adult participants to use their historical patient data (IRB approval number: 194/2006).

In this retrospective analysis fundus images of 69 T1D patients are analyzed. These images were obtained after inclusion between 1989 and 1992. Red-free and color fundus 60° images were taken with a Canon CF-60UVI EOS 35 mm film camera, Ota City Tokio, Japan (42). As digital images were not available in this study, scanned negatives were used. A variety of factors can affect the measurement of RVGC with such images. In general, the scanning resolution or compression factor plays a major role in subsequent image analysis (43). For the current analysis, the original negatives were scanned with an Epson V550 scanner. A high resolution of 9,000 × 5,300 pixels and a lossless TIFF image format were chosen.

### 2.2. Retinal vessel geometric characteristics

Analyses of retinal vessel geometric characteristics were historically carried out based on analogous fundus images and thus procedures analyzing digital images in the field today were influenced by the methodological approach of former times. For example, digital images enable pixel-based analysis of retinal vessel geometric characteristics (44–46), furthermore, pixel-to-micron conversion is common to relate current research findings to each other or to former clinical work. Cross-referencing with the literature is important to study the association between vascular parameters and systematic parameters, or disease state or risk. Comparisons between software packages on the same set of images aim to address this difficulty of methodological comparability between studies (18, 19, 28).

In the current study three types of analyses were done using Singapore Vessel I Assessment (SIVA) version 3.0 software, acquired through Exploit Technologies Pte Ltd. (Singapore) (47).

1. In accordance with SIVA standard operating procedure, ODC fundus images were used for the first analysis with SIVA with a constant image conversion factor. Variability of RVGC between the left and right eyes has previously been shown to be non-significant (48). However, in cases of differing refraction (49) or differing states of DR between the eyes, retinal parameters e.g., caliber or fractal dimension (Df) might be significantly different between eyes (50). Potential refractive error was not known in all cases in our study, and thus both eyes were analyzed with SIVA to investigate refraction-based differences between the eyes.
2. Further, to show the effect of the ICF, images of the right eye of each patient were analyzed with an individual ICF, compared with the constant ICF. If the right eye image was not available or had poor quality, the image of the left eye was used (*N* = 9).
3. To give an impression of the effect of centering (51), an analysis of both MC and ODC image of the same eye was done. If the right eye image was not available or had poor quality, the image of the left eye was used (*N* = 9). In accordance with Neubauer et al. the same vessels were chosen in both images (52).

SIVA manual (version 3.6) includes an ICF used to determine zone size and conversion factor for the diameter measurements in pixel to micrometers. It is calculated by dividing 1,800 μm with the OD diameter in pixels (29). Ideally we would have a reference diameter for our dataset but in this case we will use the default value as recommended by SIVA protocol. In the current study, to follow this conversion method, the mean OD diameter of an individual in pixels was calculated from the mean of the horizontal and vertical diameters. Individual and constant ICF share that the fixed ODs size (1,800 μm) is used as a numerator in their calculation, but they differ in their use of the denominator. The constant ICF was calculated by the division of 1,800 μm by the average OD (aOD) size of this cohort. The individual ICF, on the other hand, was calculated for each image by dividing 1,800 μm through the individual OD (iOD) size measured from that image.


$$c\,o\,n\,s\,t\,a\,n\,t\,I\,C\,F=\frac{1,800\,\mu \,m}{a\,O\,D}$$

$$i\,n\,d\,i\,v\,i\,d\,u\,a\,l\,I\,C\,F=\frac{1,800\,\mu \,m}{i\,O\,D}$$

An individual ICF is able to account for OD sizes larger or smaller than the average OD. Additionally, in everyday–clinical care multiple cameras might be used. An individual ICF could correct for the possible resolution changes and therefore enable the establishment of RVGC in a clinical setting. Refraction changes eventually taking place between multiple visits could also be corrected for with an individual ICF.

In this study, the automatic optic disk detection of SIVA did not function with the scanned negatives. However, a consistent OD edge detection process is crucial for reliable results. As automation considerably contributes to lowering inter-/intra-operator variability (53), the use of scanned fundus images without automatic OD detection is a potential source of error. To keep this error at a minimum, the OD was marked by adapting a circular template to shape along the edge of the optic nerve head prior to analysis in every image by one operator (CS). This rim was registered by SIVA in the manual mode.

Retinal vessel geometric characteristics were measured in all analyses. Central retinal artery equivalent (CRAE), central retinal vein equivalent (CRVE) and arteriovenous ratio (AVR), fractal dimension (Df), and simple tortuosity of arterioles (STa) and veins (STv) were assessed. CRAE, CRVE, and AVR were calculated with the “big six formula” by Knudtson et al. (54), which calculates the central equivalent of the six largest arterioles and veins in zone B and C. A conversion from the pixel measurement of vessels to μm is done by multiplying the ICF with the pixel count.


C⁢R⁢A⁢E⁢i⁢n⁢μ⁢m=I⁢C⁢F×C⁢R⁢A⁢E⁢i⁢n⁢p⁢i⁢x⁢e⁢l

**FIGURE 1:**
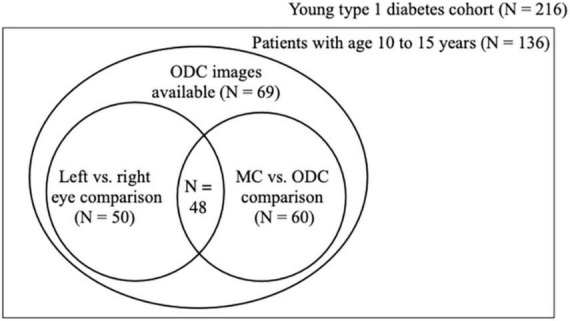
Population set up. Schematic showing how the three analyses relate to each other in the cohort material. Of the 69 patients 50 had ODC images taken of both the left and the right eye and 60 had ODC and MC images of the right eye. ODC, optic disk-centered fundus images; MC, macula-centered images.

The larger zone C spans from half of the OD margin to double its diameter. In zone C a box counting algorithm calculated Df (55). Total simple tortuosity (STt), an extended CRAE (CRAEoC), as well as CRVE and AVR were also calculated in zone C (CRVEoC, AVRoC). The tortuosity measurement used was simple tortuosity, which is calculated from the ratio of the tracked path length of a vessel segment to the straight-line length of the segment (34). See Figure 1 for the width of the zones.

### 2.3. Statistical analysis

For descriptive statistics constant variables are presented by their mean ± standard deviation and categorical variables will be presented by their count and percent of the total.

Two-sided paired samples *T*-test was used for normally distributed data and a related samples Wilcoxon signed rank test was employed for positively or negatively skewed data to determine mean (md) or median differences between the RVGC calculated in the three analyses. To compare employed analysis methods, Bland-Altman (56) mean difference was calculated between ODC individual ICF (iICF) minus ODC with constant ICF (cICF) and ODC iICF minus MC iICF, respectively. To investigate systematic bias, we used one sample *T*-test, comparing the mean difference and zero value. For proportional bias linear regression was used with the mean difference as dependent variable and the mean of analyzed methods as independent variable. The slope of this regression line was compared to a zero value with one sample *T*-test.


d=R⁢V⁢C⁢G⁢w⁢i⁢t⁢h⁢i⁢I⁢C⁢F-R⁢V⁢C⁢G⁢w⁢i⁢t⁢h⁢c⁢I⁢C⁢F

$$m\,d=\frac{\Sigma \,\text{d}}{N}$$

For multiple comparisons concerning the twelve RVGC, the significance threshold was Bonferroni-corrected. To obtain the Bonferroni-corrected *p*-value, we divided the original α-value by the number of analyses on the dependent variable. The Bonferroni-corrected *p*-value was considered statistically significant when <0.0042.

Testing of the association of medical characteristics with the measured RVGC was important, as there is no ground truth when it comes to sizes measured in fundus images. Pearson correlation values were calculated to examine the relationship between medical characteristics and retinal vessel characteristics. Multivariable linear regression with stepwise inclusion of independent variables (RVGC) was used to find the best suited model for individual medical characteristics. The statistical analysis was performed with SPSS (version 24.0, SPSS Inc., Chicago, IL, USA) and Python 3.7.4. Available at http://www.python.org. (Python Software Foundation, Beaverton, OR, USA).

## 3. Results

### 3.1. Study population

Optic disk centered (ODC) photographs of 69 children with T1D were examined. The mean age of the patients was 12.2 ± 1.6 years, with a mean diabetes duration of 5.9 ± 3.0 years. 49% (*n* = 34) were male. Seven children had early or mild DR and one child had moderate DR. The clinical characteristics are presented in Table 2.

**TABLE 2:** Mean and standard deviation of clinical characteristics of the cohort, as available.

Clinical characteristics (*N*)	Mean ± SD	Normal ranges (64)
Age, years (69)	12.2 ± 1.6	
T1D duration, years (69)	5.9 ± 3.0	
Insulin dose, Units/kg (51)	0.75 ± 0.2	
Hb, g/L (69)	135.7 ± 7.8	115–140
Creatinine, μmol/L (69)	55.1 ± 10.5	40–90
HbA1, % of total Hb (60)	12.3 ± 2.4	<8.0
Total cholesterol, mmol/L (63)	4.4 ± 0.7	2.9–6.0
Thyroid-stimulating hormone, mU/L (51)	2.3 ± 1.5	0.5–4.5
Systolic BP, mmHg (50)	113.3 ± 8.9	100–124 (64)
Diastolic BP, mmHg (50)	70.7 ± 8.7	65–79 (38)

Where suitable, normal ranges are given. BP, blood pressure; Hb, blood hemoglobin; HbA1, glycated hemoglobin; T1D, type 1 diabetes.

### 3.2. Retinal vessel geometric characteristics

The feature for automatic OD detection did not function in any of the three analyses with the scanned images of this cohort. Both ODC and MC images presented sufficient number of vessels for calculation of central retinal equivalents according to the “big six-formula” (54).

As the next step, SIVA detected the vessels. Accurate detection of vessels and vessel types was manually checked in all cases. The vessel type (e.g., artery or vein) of one or two vessel branches also had to be manually corrected in most cases. If less than six vessels were detected in the first processing - typically it was five or four - marking of vessels were added manually to meet the total of six. The duration of the analysis of a single image was approximately 5 min. Image quality did not allow for reliable vessel type classification in two of 69 cases, and analysis for those was thus not possible.

**FIGURE 2:**
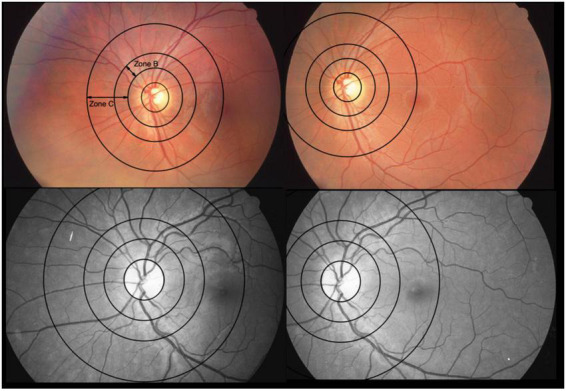
Optic disk-centered and macula-centered photographs of the same eye of two patients. The color fundus photographs are from one patient and the red-free-photos from another. They represent 2 extremes with differences between central retinal vein equivalents (CRVE) for use of constant image correction factor (cICF) and individual ICF of 62 μm (color) and –67 μm (red-free), respectively. The mean of the CRVEs from the 2 measurements 191 μm for the color photo and 199 μm for the red-free photo. Zone B is 0.5 to 1 OD diameter wide. Zone C spans from 0.5 to 2 OD diameters. The 2 extremes can also be found in the Bland-Altman plot for CRVE in Figure 5.

As not all patients had fundus photographs taken from their left and right eye or ODC and MC, fifty patients had ODC images of both eyes (first analysis). The buildup of the cohort and the analyses performed are illustrated in Figure 2.

The mean calculated CRAE with standard deviation resulting from the first analysis with the constant ICF of 2.4 was 162 ± 14.4 μm, CRVE was 209 ± 19.0 μm, and mean Df was 1.24 ± 0.05.

**FIGURE 3:**
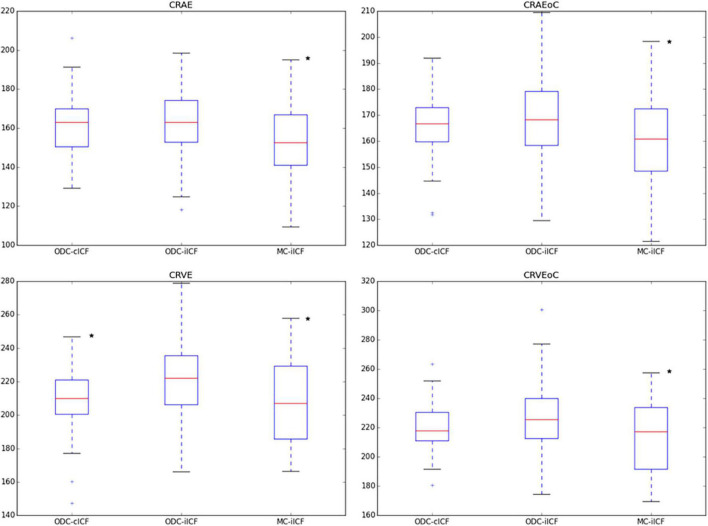
Box plots for results of central retinal equivalents. The box plots show the results for the central retinal equivalents CRAE and CRVE for zone B and zone C (CRAEoC and CRVEoC) for the analysis of optic disk centered (ODC) fundus photographs with a constant image conversion factor (cICF) and individual ICF (iICF) and of macula centered (MC) images with iICF. The y-axis is in μm and the exact mean ± standard deviation can be found in the Supplementary Table 1. Two-sided paired samples *T*-test or related samples Wilcoxon signed rank test were used for the comparison of the different methods. The significance threshold was Bonferroni-corrected to <0.0042. *Significant values. CRAEoC, central retinal artery equivalent of zone C; CRVEoC, central retinal vein equivalent of zone C.

**FIGURE 4:**
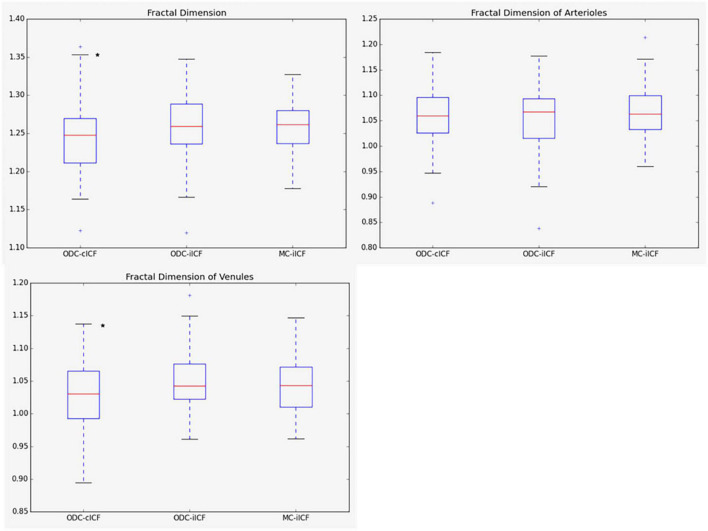
Box plots for results of fractal dimension. The box plots show the results for the fractal dimension (Df) for the analysis of optic disk centered (ODC) fundus photographs with a constant image conversion factor (cICF) and individual ICF (iICF) and of macula centered (MC) images with iICF. The y-axis is without units and the exact mean ± standard deviation can be found in the Supplementary Table 1. Two-sided paired samples *T*-test or related samples Wilcoxon signed rank test were used for the comparison of the different methods. The significance threshold was Bonferroni-corrected to <0.0042. *Significant values.

In the second analysis the ICF was adjusted individually (iICF). The mean individual ICF was 2.4 ± 0.18. The resulting mean CRAE was 163 ± 15.6 μm, mean CRVE was 219 ± 21.8 μm, and mean Df was 1.26 ± 0.04. The third analysis also used an individual ICF for the comparison of MC and ODC images. The mean iICF here was 6.2 ± 0.58 for the MC images. The RVGC results for the comparison of right eye with constant ICF and individual ICF, as well as the results for the MC images are shown the Figures 3, 4 and Supplementary Figures 1, 2. The exact results can be found in the Supplementary Table 1. Table 3 shows the results for the analysis of both eyes with constant ICF.

**TABLE 3:** Results of retinal vessel geometric characteristics (RVGC) comparing left and right eye with SIVA.

RVGC	Constant ICF, right eye	Constant ICF, left eye	Left vs. right eye, *p*-value (*N* = 50)
CRAE, μm	162 ± 14.4	162.6 ± 16.4	0.900
CRVE, μm	208 ± 19.0	208 ± 21.7	0.934
AVR	0.78 ± 0.096	0.79 ± 0.103	0.444
CRAEoC, μm	167 ± 13.2	169 ± 14.9	0.463
CRVEoC, μm	218 ± 14.6	218 ± 17.8	0.924
AVRoC	0.76 ± 0.070	0.78 ± 0.083	0.502
Df	1.25 ± 0.045	1.25 ± 0.054	0.866
Dfa	1.07 ± 0.062	1.06 ± 0.083	*0.377*
Dfv	1.03 ± 0.053	1.02 ± 0.070	*0.813*
STt	1.09 ± 0.020	1.09 ± 0.020	*0.295*
STa	1.10 ± 0.033	1.10 ± 0.034	*0.949*
STv	1.08 ± 0.015	1.08 ± 0.015	*0.629*

The first analysis involved optic disk centered (ODC) fundus images from right and left eye of a participant with a constant image conversion factor (ICF). The RVGC are given by their mean ± standard deviation. Two-sided paired samples *T*-test or related samples Wilcoxon signed rank test (results for skewed variables in italic font) were used for the comparison of the different methods. The significance threshold was Bonferroni-corrected to < 0.0042. CRAEoC, central retinal artery equivalent of zone C; CRVEoC, central retinal vein equivalent of zone C; AVRoC, arteriovenous ratio of zone C; Df, fractal dimension; STt, total simple tortuosity; *a*, values summarized for arteries; *v*, values summarized for veins.

The paired samples *T*-test and related samples Wilcoxon signed rank test comparing the right and left eyes in the first analysis turned out to be non-significant for any of the measured parameters (Table 3). A continuation with only one eye per patient in second analysis was thus justified. The paired samples *T*-test and related samples Wilcoxon signed rank test for the MC-ODC comparison was significant for the diameter measurements (CRAE *p*-value = 0.001, CRVE *p*-value = 0.001) (Supplementary Table 1).

Table 4 was based on the Supplementary Table 4 from McGrory et al. showing the difference between a constant/average ICF and an individual ICF (28). It gives an impression on how much the caliber values in μm can differ, depending on the ICF used. The paired samples *T*-test remained non-significant for the diameter measurements of the CRAE. The smallest ICF 1.78 has the narrowest corresponding central retinal equivalents (CRAE = 124 μm, CRVE = 166 μm), while the biggest ICF 2.82 produces the widest measurements (CRAE = 188 μm, CRVE = 272 μm).

**TABLE 4:** Comparing the diameter measurements calculated with a constant image conversion factor (cICF = 2.4) and an individual ICF.

				CRAE in μ m			CRVE in μ m		
	OD diameter in pixels	iICF formula	ICF	With iICF	With cICF	Difference	With iICF	With cICF	Difference
1st	638.0	1,800/6,38.0	2.82	188	163	25	272	236	36
quintile	693.2	1,800/6,93.2	2.61	159	149	10	191	179	12
2nd	693.2	1,800/6,93.2	2.61	181	173	8	246	236	10
quintile	722.0	1,800/7,22.0	2.49	147	144	3	233	228	5
3rd	722.0	1,800/7,22.0	2.49	183	179	4	245	240	5
quintile	748.6	1,800/7,48.6	2.41	154	156	−2	223	226	−3
4th	748.6	1,800/7,48.6	2.41	162	165	−3	206	210	−4
quintile	782.0	1,800/7,82.0	2.3	151	159	−8	195	26	−11
5th	782.0	1,800/7,82.0	2.3	150	159	−9	193	205	−12
quintile	1014.0	1,800/1,014.0	1.78	125	172	−47	166	228	−62

The average of the individual ICF (iICF) was 2.4. CRAE, central retinal artery equivalent; CRVE, central retinal vein equivalent, both of zone B.

**FIGURE 5:**
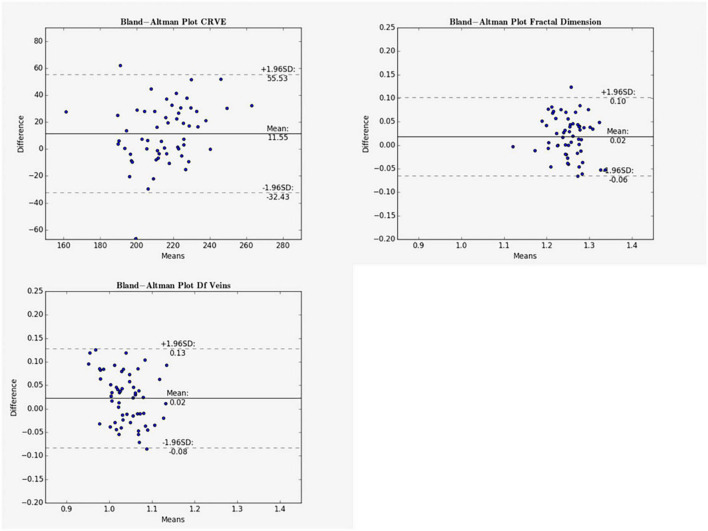
Bland-Altman plots for vein measurements comparing cICF and iICF. The Bland-Altman plots show the results for central retinal equivalents of veins (CRVE), fractal dimension (Df) and fractal dimension of veins for the analysis of optic disk centered (ODC) fundus photographs with a constant image conversion factor (cICF) and individual ICF (iICF). The x- and y-axes are in μm and the exact values can be found in the Table 5. The significance threshold was Bonferroni-corrected to <0.0042. *Significant values.

**FIGURE 6:**
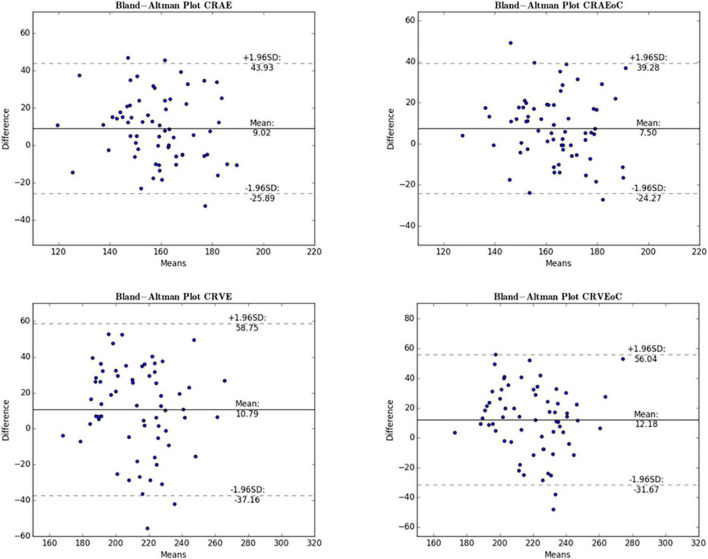
Bland-Altman plot for central retinal equivalents comparing ODC and MC. The Bland-Altman plots show the results for the central retinal equivalents of arteries (CRAE) and veins (CRVE) for the analysis of optic disk centered (ODC) fundus photographs with an individual image conversion factor (iICF) and of macula centered (MC) images with an iICF. The x- and y-axes for CRVE are in μm and the exact values can be found in the Table 5. The significance threshold was Bonferroni-corrected to <0.0042. *Significant values.

Mean differences between ODC individual ICF versus constant ICF was investigated with Bland-Altman analysis, as seen in Table 5. The individual ICF RVGC are more positive, resulting in a positive mean difference for most investigated parameters. CRVE, Df and Dfv are significantly different as shown in Figure 5. In Table 6, the Bland-Altman analysis for ODC individual ICF versus MC individual ICF is shown. Generally, the RVGC from ODC individual ICF are more positive than MC individual ICF. Central retinal equivalents are statistically significant, as the point of view has shifted (Figure 6). Plots of the non-significant Bland-Altman results are depicted in Tables 5, 6 and are displayed in Supplementary Figures 3–6.

**TABLE 5:** Bland-Altman analysis for optic disk centered (ODC) individual image conversion factor (ICF) versus ODC constant ICF.

RVGC	Mean difference	Std. deviation	Upper limit	Lower limit	One sample *T*-Test	Linear regression
CRAE	2.7	18.02	38.00	-32.63	0.269	0.451
CRAEoC	1.4	15.68	32.14	-29.32	0.505	0.191
CRVE	11.6	22.44	55.53	-32.43	<0.001*	0.070
CRVEoC	7.5	22.78	52.11	-37.20	0.018	0.003*
AVR	-0.03	0.0910	0.1499	-0.2071	0.014	0.002*
AVRoC	-0.02	0.0713	0.1249	-0.1601	0.051	0.026
Df	0.0192	0.0450	0.1074	-0.0690	0.001*	0.190
Dfa	0.0006	0.0699	0.1376	-0.1364	0.946	0.650
Dfv	0.0212	0.0554	0.1297	-0.0873	0.004*	0.052
STt	0.0015	0.0160	0.0328	-0.0298	0.470	0.623
STa	0.0042	0.0223	0.0480	-0.0396	0.150	0.209
STv	-0.0005	0.0160	0.0309	-0.0319	0.795	0.582

One sample *T*-test tested the mean difference against 0. Statistically significant linear regression would indicate proportional bias. The significance threshold was Bonferroni-corrected to <0.0042. Graphical representation of the Bland- Altman results can be found in Figure 5 and Supplementary Figures 3, 4. RVGC, retinal vessel geometric characteristics; CRAEoC, central retinal artery equivalent of zone C; CRVEoC, central retinal vein equivalent of zone C; AVRoC, arteriovenous ratio of zone C; Df, fractal dimension; STt, total simple tortuosity; *a*, values summarized for arteries; *v*, values summarized for veins. *Significant values.

**TABLE 6:** Bland-Altman analysis for optic disk centered (ODC) individual image conversion factor (ICF) versus macula-centered (MC) individual ICF.

RVGC	Mean difference	Std. deviation	Upper limit	Lower limit	One sample *T*-Test	Linear regression
CRAE	9	17.8	43	-26	<0.001*	0.214
CRAEoC	8	16.2	39	-24	<0.001*	0.312
CRVE	11	24.5	59	-37	<0.001*	0.290
CRVEoC	12	22.4	56	-32	<0.001*	0.330
AVR	0.004	0.091	0.1887	-0.1757	0.610	0.281
AVRoC	-0.008	0.075	0.1415	-0.16	0.400	0.258
Df	0.0012	0.035	0.0719	-0.0701	0.785	0.198
Dfa	-0.014	0.062	0.1104	-0.1404	0.075	0.033
Dfv	0.0071	0.063	0.13	-0.12	0.375	0.935
STt	0.0022	0.0124	0.0261	-0.0229	0.161	0484
STa	0.0033	0.0188	0.0384	-0.0338	0.170	0855
STv	0.0013	0.0155	0.0335	-0.0300	0.499	0.536

One sample *T*-test tested the mean difference against 0. Statistically significant linear regression would indicate systematic bias. The significance threshold was Bonferroni-corrected to <0.0042. Graphical representation of the Bland- Altman plots can be found in Figure 6 and Supplementary Figures 5, 6. RVGC, retinal vessel geometric characteristics; CRAEoC, central retinal artery equivalent of zone C; CRVEoC, central retinal vein equivalent of zone C; AVRoC, arteriovenous ratio of zone C; Df, fractal dimension; STt, total simple tortuosity, *a*, values summarized for arteries; *v*, values summarized for veins. *Significant values.

Cholesterol positively correlated significantly with Df (Pearson-rho = 0.375, *p*-value = 0.008, number = 49) and STv (0.297, 0.038, 49). The duration of diabetes correlated with the total (0.410, 0.002, 53) and artery tortuosity (0.375, 0.006, 53) measurements, but also with AVRoC (0.304, 0.027, 53) and CRAE 0.284, 0.040, 53). However, clinical parameters that correlated with a RVGC calculated with an individual ICF did not necessarily correlate with a vessel parameter calculated with a constant ICF or when a MC image was used. For example, CRAEoC for individual ICF correlated with duration (0.275, 0.046, 53) but not when using the other two analysis methods. Other times two out of three analyses correlated, e.g., Dfv and HbA1 for constant (−0.369, 0.014, 44) and individual (−0.379, 0.011, 44) ICFs. STt, STv and duration of diabetes showed consistent correlation between the analysis methods (STt -constant ICF: 0.373, 0.006; MC: 0.433, 0.001, STv-constant ICF: 0.307, 0.0025, MC: 0.383, 0.005 *N* = 53 for all) (Table 7).

**TABLE 7:** Pearson correlation results between retinal vessel geometric characteristics (RVGC) and clinical parameters.

Medical characteristics (*N*)	RVGC	Constant ICF	Individual ICF	Macula-centered
Serum	Df	0.214, 0.140	0.375, 0.008	0.342, 0.016
Cholesterol (49)	Dfa	0.211, 0.146	0.365, 0.010	0.256, 0.075
	STv	0.113, 0.440	0.297, 0.038	0.030, 0.840
Hb (53)	Df	−0.069, 0.621	0.014, 0.919	−0.307, 0.025
Serum creatinine (53)	STa	0.119, 0.397	0.254, 0.066	0.266, 0.040
T1D duration (53)	CRAE	0.172, 0.217	0.284, 0.040	0.056, 0.690
	CRAEoC	0.123, 0.381	0.275, 0.046	0.037, 0.795
	AVR	0.099, 0.480	0.282, 0.040	−0.039, 0.782
	AVRoC	0.190, 0.174	0.304, 0.027	0.030, 0.832
	STt	0.373, 0.006	0.410, 0.002*	0.433, 0.001*
	STa	0.307, 0.0025*	0.375, 0.006	0.383, 0.005
HbA1 (44)	Dfa	0.052, 0.739	0.336, 0.026	−0.190, 0.217
	Dfv	−0.369, 0.014	−0.379, 0.011	0.078, 0.614
	STt	0.395, 0.008	0.291, 0.055	0.248, 0.104
	STv	0.412, 0.005	0.185, 0.229	0.268, 0.079
HbA1 ‘83–‘89 (48)	Dfv	−0.191, 0.193	−0.335, 0.020	0.062, 0.677
Insulin dose (38)	CRAEoC	−0.231, 0.163	−0.051, 0.761	−0.425, 0.008
	AVRoC	0.107, 0.523	0.156, 0.349	−0.316, 0.037
	Df	−0.364, 0.025	−0.219, 0.187	−0.392, 0.015
Signs of DR (53)	Dfv	−0.342, 0.012	−0.258, 0.062	0.028, 0.841
Systolic BP (37)	CRVE	−0.017, 0.922	−0.031, 0.858	0.341, 0.039
	AVR	−0.045, 0.791	0.093, 0.583	−0.406, 0.013
	STa	0.340, 0.040	0.244, 0.146	−0.307, 0.065
Diastolic BP (37)	STa	0.329, 0.047	0.270, 0.106	0.315, 0.058
State of puberty (37)	AVR	−0.144, 0.394	−0.110, 0.518	−0.391, 0.017
	AVRoC	−0.386, 0.018	−0.054, 0.751	−0.343, 0.037

Pearson correlation was calculated for sub-samples of the cohort that had to have the clinical parameter investigated, RVGC measurements from ODC images with constant ICF and individual ICF, as well as MC images with individual ICF to be included in the analysis. Results are given in order of Pearson coefficient rho, *p*-value. The significance threshold was Bonferroni-corrected to <0.0042. AVRoC, arteriovenous ratio of zone C; CRAEoC, central retinal artery equivalent of zone C; CRVEoC, central retinal vein equivalent of zone C; Df, fractal dimension; ICF, image conversion factor; MC, macula centered; ODC, optic disk centered; RVGC, retinal vessel geometric characteristics; STt, total simple tortuosity; *a*, values summarized for arteries; *v*, values summarized for veins *p-value <0.0042.

Leading on from the correlation presented in Table 7, multivariable linear regression with step-wise inclusion was performed in Table 8. RVGC variables, calculated with a constant ICF were included to a model for the following medical characteristics: serum creatinine, Hb1A, insulin dose. Serum cholesterol, T1D Duration, HbA1 and HbA1 for ‘83–‘89 had RVGCs calculated with individual ICF added to their multivariable linear regression. RVGC calculated with an individual ICF computed from MC images were included to a model for the regression of serum cholesterol, Hb, serum creatinine, insulin dose, and systolic and diastolic BP.

**TABLE 8:** Multivariable linear regression with stepwise inclusion of retinal vessel geometric characteristics (RVGC) as independent variables.

Medical characteristics (*N*)	RVGC	Constant ICF	Individual ICF	Macula-centered
Serum Cholesterol (51)	Df		0.342 (1.22–10.48), 0.014	0.326 (1.42–12.23), 0.014
Hb (55)	Df			−0.306 (−121.75 to −12.14), 0.018
Serum creatinine (55)	STa	0.298 (9.91–160.35), 0.027		0.266 (4.15–165.52), 0.040
T1D Duration (55)	CRAE		0.250 (0.02–0.095), 0.040	
HbA1 (46)	Dfa		0.472 (8.75–30.78), 0.001	
	Dfv	−0.597 (−51.68 to −13.77), 0.001	−0.547 (−45.43 to −15.95), <0.001	
	CRVEoC	0.385 (0.01–0.09), 0.030		
HbA1 ‘83-‘89 (50)	Dfv		−0.366 (−29.37 to −4.31), 0.009	
Insulin dose (40)	CRAEoC			−0.322 (−0.01 to 0.00), 0.033
	Dfa	−0.404 (−1.93 to −0.29), 0.010		
Systolic BP (39)	AVR			−0.373 (−86.1 to −12.6), 0.010
	Dfv			−0.348 (−115.4 to −12.9), 0.015
Diastolic BP (39)	STt			0.322 (8.7–233.4) 0.035

RVGC were calculated with a constant image conversion factor (ICF), an individual ICF and with an individual ICF from Macula-centered images. Results are given as beta (confidence interval), *p*-value. Free spaces indicate that no RVGC was added to the model. Hb, blood hemoglobin; T1D, type 1 diabetes; HbA1, glycated hemoglobin; BP, blood pressure; RVGC, retinal vessel geometric characteristics; CRAEoC, central retinal artery equivalent of zone C; CRVEoC, central retinal vein equivalent of zone C; AVRoC, arteriovenous ratio of zone C; Df, fractal dimension; STt, total simple tortuosity; *a*, values summarized for arteries; *v*, values summarized for veins.

## 4. Discussion

The analysis of fundus images in this study displays several challenges in connection with commonly used vessel assessment software. We present the effect of changed image modalities, variable image centering and use of ICF on measured RVGC. Problems were identified in the use of constant versus individual ICF application, changed centering of the image (ODC vs. MC) and its effect on clinical associations. The commonly used OD diameter used for ICF calculation influences the vessel diameter measurement given in μm.

As the individual ICF’s differ from the constant ICF used in the first analysis, individual diameter measurements end up being either narrower or wider. This depends on the individual ICF being smaller or bigger than the constant ICF. Therefore, the diameter measurements of the individual ICF analysis are over-all wider on average than in the first analysis but only statistically significant for the CRVE (Figure 3 and Supplementary Table 1). The same could be observed in Figure 5 and Table 5, were the Bland-Altman analysis found poor agreement with more positive mean differences for the CRVE measurements when comparing measurements done with individual ICF vs. constant ICF. This means that the constant ICF measures statistical significantly smaller values for central retinal vein equivalents (mean difference of 11.6 and 7.5 μm), a trend for smaller values was also observed for central retinal artery equivalents. For arteriovenous ratios, fractal dimension and simple tortuosity, measurements with individual ICF were up to 0.03 times smaller, which was negligible. Numerical values can be obtained from Table 6 and plots can be found in Figure 5 and Supplementary Figures 3, 4. As can be seen in the box plots of Figures 3, 4 or Table 4, values that were obtained using an individual ICF are more likely realistic, showing a larger range of vessel diameters. To establish individual wellbeing of the vasculature, a constant ICF is not sufficient, because it bases its calculation on the average OD size of a cohort. Instead, the individual ICF factors in differences of OD of each eye to produce a risk assessment of a particular patient.

In the past (57), the average grid was converted to determine the size of the measurement zones. Now, with software, the ICF determines the mean zone size (28). An average ICF is usually calculated from the mean OD size of a sizable percentage (10% with SIVA) of the cohort or the mean optic disk diameter is computed from the whole set of images (19, 28). The calculation of the ICF is facilitated by an average OD diameter instead of raw pixel measurements of individual images adjusted for ocular magnification of the eye. The use of a fixed micron reference for OD for any cohort is a source of variation or error influencing caliber measurements. Optic disk size can vary significantly between subjects. Quigley et al. and others (58, 59) found that ethnicity and sex can affect OD size. For a Finnish or even Scandinavian cohort no reference measure for OD size was previously established. Generally, using a constant conversion factor (especially if the 1,800 μm is not a true reference diameter for the cohort) is particularly problematic for individuals with extreme optic disk diameter or if the cohort had considerable spread in OD diameter. This can be, arguably, more inconvenient than leaving the measurement in the original units of pixels.

Generally, a large ICF indicates a small OD diameter and therefore a smaller measurement zone. Hereby, vessels measured are nearer to the OD and therefore anatomically have the tendency to be wider. A small ICF indicates a wider OD diameter, which in turn widens the measurement zone. With this, vessels from farther away from the OD are included into the calculations, therefore, smaller ICFs have the tendency to be associated with narrower diameter measurements. Figure 2 illustrates this. In Table 4 this trend can be observed when looking at the columns of vessel diameters in μm.

Diameter measurements done with a constant ICF are limiting the variance of the RVGC results, artificially enlarging or shrinking values depending on the individual ICF, which can either be larger or smaller than the constant ICF in any individual eye. CRVE and CRAE computed with individual ICF are a better estimate for individual measurements, even if the variance within the cohort increases. Vessel assessment software users should be aware of this.

Generally, an individual ICF presents the individual range of caliber measurements, as can be observed in McGrory’s Supplementary Table 4 (28). In their study the difference between constant and individual diameter measurements was significant. In the current study not all results were statistically significantly, which could stem from a smaller difference between average individual and constant ICF. An individual ICF leads to different measurement values of the vessel diameters in μm. Its apparent non-use could be explained by the necessity to calculate a new ICF of every photograph. Future automation can remedy that. To avoid problems caused by conversion of pixel diameter to μm diameters with ICF, one could directly use the calculated central retinal equivalents measurements in pixel (17, 28). However, an approximation of width in μm helps clinical judgment, and by using pixels a comparison to existing studies would be hindered. A further option for utilizing central retinal equivalent for individual disease progression could be to establish a ratio for change between visits, which is only possible when there is no change in camera or if a correction factor can be obtained.

In a clinical setting RVGC are best measured by a trained technician. Acquiring RVGC with any software is likely to involve the knowledge of camera settings and other information to produce reliable results. In the future, there might be fully automated software with input from the fundus camera, which would produce for results simply by clicking a button. For now, RVGC analysis is not standardized enough to produce comparable results between studies (18, 20).

In this study the comparison of MC and ODC images gave statistically significant results for the central retinal equivalents (Figure 3 or Supplementary Table 1) with poor agreement from Bland-Altman analyses (Figure 6 or Table 6), this is comparable to Mookiah et al. (51). Even though all RVGC-software recommend the use of ODC images, some studies only have MC images available (26, 60). Therefore, it was paramount to find out how the centering is related to the characteristics measured. In the Bland-Altman analysis for ODC vs. MC images we found that RVGC were generally larger measured from ODC images, compared to MC images (Table 6) (camera and resolution settings were kept the same). Therefore, future studies using MC images need to keep in mind that RVGC might be smaller in comparison to ODC images, or vice versa. In the case of our 60° images, only the vessel caliber results are affected significantly (Figures 3, 6 or Supplementary Table 1 and Table 6). The lack of agreement for the caliber measurements could also be caused by zone C being cut off to a varying extent in MC images, as can be seen in Figure 2. In the past, diameter measurements were shown to be more easily affected by changed image modalities and different analysis software (18). Also, their correlation coefficients with clinical characteristics can change (28). In this study AVR were identical between MC and ODC images (Table 6 and Supplementary Figure 5), with Bland-Altman analysis indicating negligible differences. This indicates that a factor between ODC and MC cancels itself out when it is derived from CRAE and CRVE. Therefore, despite variable image centering, measurements can be directly compared between ODC and the 60° MC images employed in this study, where Zone C is complete in both points of view. Generally, the AVR is a more robust characteristic when it comes to measurement changes due to resolution, angle, or calculation technique (49, 52), but important information about exact changes in arteriolar and venous vessels is lost (61).

Measurements of fractal dimension and simple tortuosity also showed good agreement in the Bland-Altman analysis, proving that they are similar despite different image centering. This again indicates that a factor between ODC and MC cancels itself out when Df and ST are computed and therefore such derived measurements can be directly compared between MC and ODC. Numerical values can be obtained from Table 6 and plots can be found in Supplementary Figures 5, 6.

Individual ICF allows for a more flexible inclusion of different image modalities, this is also possible for single patients. As mentioned before, the same vessels need to be selected to always make analysis methods comparable. Generally, if MC images are used to analyze RVGC it is important to select images showing a larger degree of the retina, including at least zone B and most of zone C. Preferably, vessels included in the analysis are not on the edge of the image to avoid possible distortion. Therefore, studies using MC images need to take the above into account, when deriving RVGC. This warning applies to macula centered images with 45° or 30° field of view, for example, where Zone C can be substantially incomplete. In these cases vessels detected would be reduced and the results of the calculations could be affected.

Generally, diameter measurements show association with clinical characteristics (15), for example, Broe et al. measured the vessel caliber in an adolescent T1D cohort and found correlation between diabetes duration, HbA1c and CRVE, and CRAE with systolic and diastolic blood pressure (33). The correlation coefficients of RVGC with clinical parameters of this study differed between the three analysis methods (Table 7). For example, the duration of diabetes correlated with six RVGCs measured with an individual ICF and only with two RVGC measured with a constant ICF and only with two from MC images. Part of this, the STt and Sta were the only characteristics to be statistically significant correlated through all analysis methods with duration of diabetes. The positive correlation of STt and STa indicated that a longer duration results in more curved arteries (62, 63). After Bonferroni correction only the correlation between diabetes duration and STt calculated with individual ICF for ODC and MC images persisted. Associations evaluated with multivariable linear regression also differed between the analysis methods (Table 8), however, testing their effect on associations with systemic variables is outside the scope of this manuscript. Contrary to prior studies involving adults with diabetes (64), the caliber measurements (calculated with constant ICFs in this study) showed no statistically significant correlation with clinical characteristics. This is not surprising, as this study consists of children who were of good health except for a still relatively short duration of diabetes and only a few with even mild retinopathy.

## 5. Conclusion

Scanned fundus images can be analyzed with modern tools. This is possible when using an individual ICF for analyzing ODC images or 60° MC images. RVGC, except for central retinal equivalents can be used to assess the vasculature of eyes. The results of the three analyses illustrate the challenges of establishing normal and abnormal values of RVGC in a clinical setting. On top of the general limitations of employing a constant reference OD for pixel-to-micron conversion, the analyses with constant and individual ICF result in differing values for retinal caliber measurements in μm, e.g., a refractive error outside the norm will affect central equivalent measurements disproportional with a constant ICF compared to an individual ICF. As shown by the 60° images of the current work, where Zone C is complete in both points of view, the analysis of MC and ODC images resulted in good agreement for arteriovenous ratio, fractal dimension and simple tortuosity measurements, signaling that MC and ODC images could be used interchangeably when Zone C is complete. However, CRAE and CRVE are statistically significant, presenting further evidence of the susceptibility of diameter measurement to changed imaging settings. In summary, we recommend using ODC images for Zone C analysis, but if not available, MC images can be utilized under the recommendations above.

This study enhances the awareness that central retinal equivalent values calculated with different vessel assessment software or between different studies are not by default interchangeable. For evaluating the relationship of changed analysis methods on systemic associations with RVGC, a larger publicly available image and medical data base is needed. This could facilitate enhanced collaboration in the field to produce comparable individual RVGC risk values between studies.

## Data availability statement

The images analyzed for this study can be found in the health atlas repository (https://health-atlas.de/projects/43), where access to the images can be requested after registration with the health atlas.

## Ethics statement

The studies involving human participants were reviewed and approved by the Ethics Committee of the Oulu University Hospital. The patients/participants provided their written informed consent to participate in this study.

## Author contributions

CS, AF, and NH planned the study. CS scanned the images and used SIVA for analyzing the RVGC. CS and FGR did statistical analyses. All authors wrote and revised the manuscript.
