# Supplementary material for: The impact of the image conversion factor and image centration on retinal vessel geometric characteristics
Source: Front Med (Lausanne). 2023 Mar 17;10:1112652. doi: 10.3389/fmed.2023.1112652 (PMC10063888; doi:10.3389/fmed.2023.1112652)
Supplement: Supplementary file 1 [file Data_Sheet_1.pdf]

Supporting Information

to

The relationship of the image conversion factor and centering on retinal vessel geometric characteristics

Carolin Schanner [1,2], Nina Hautala [1], Franziska G. Rauscher [2], Aura Falck [1]

[1] Department of Ophthalmology and Medical Research Center, Oulu University Hospital and PEDEGO Research Unit, University of Oulu, P.O. Box 21, 90029 OYS, Finland,

[2] Institute for Medical Informatics, Statistics, and Epidemiology, Leipzig University, Haertelstrasse 16-18, D-04107, Leipzig, Germany

Corresponding author:

Aura Falck, (M.D.)

Department of Ophthalmology and Medical Research Center, Oulu University Hospital and PEDEGO Research Unit, P.O. Box 21, 90029 OYS

University of Oulu, Finland,

Email address: Aura.falck@oulu.fi

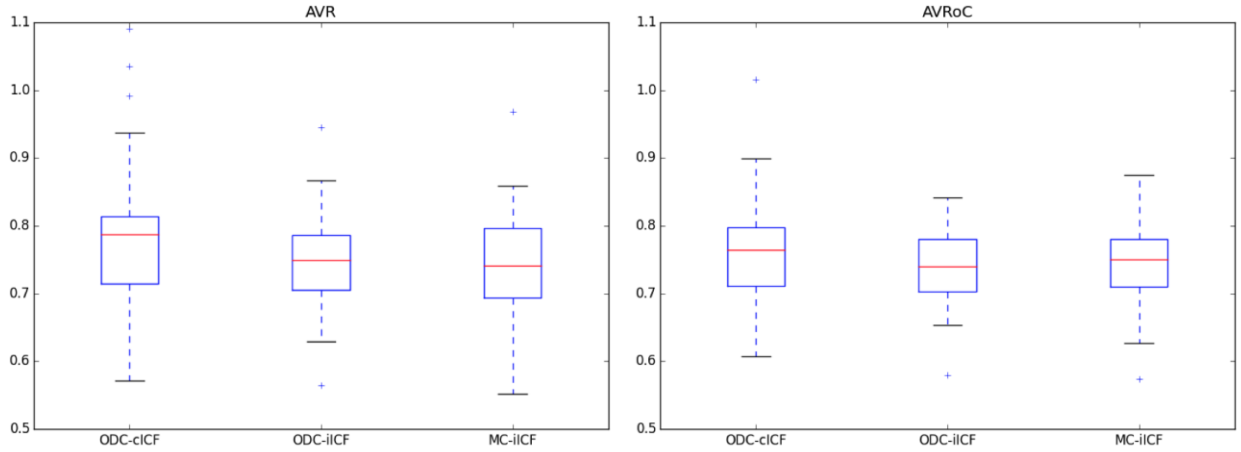

Figure S1: Box plots for results of arteriovenous ratio. The box plots show the results for the arteriovenous ratio for zone B and zone C (AVRoC) for the analysis of optic disk centered (ODC) fundus photographs with a constant image conversion factor (cICF) and individual ICF (iICF) and of macula centered (MC) images with iICF. The y-axis is without unit and the exact mean ± standard deviation can be found in the supplement material Table S1. Two-sided paired samples T-test or related samples Wilcoxon signed rank test were used for the comparison of the different methods. The significance threshold was Bonferroni-corrected to < 0.0042.

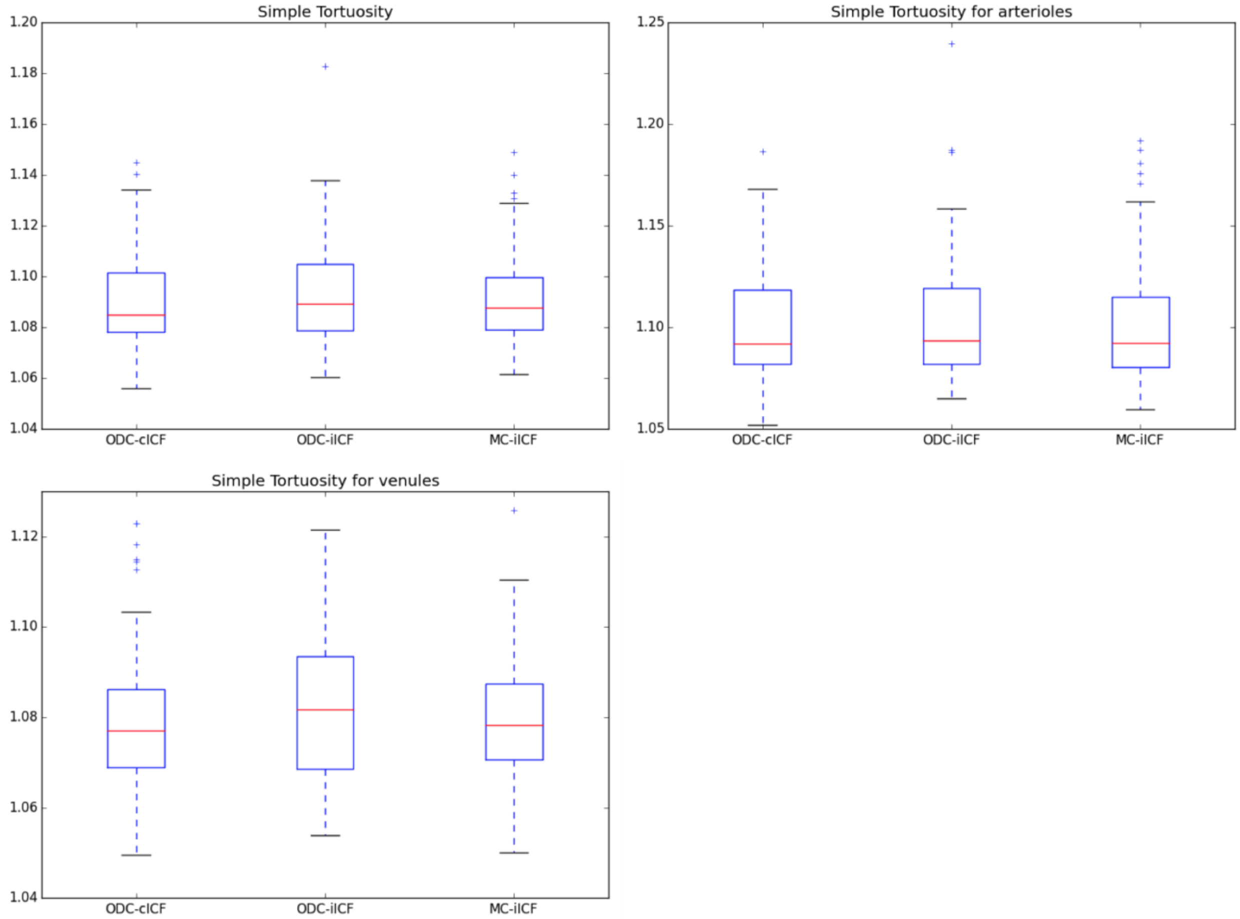

Figure S2: Box plots of results of simple tortuosity The box plots show the results for simple tortuosity for the analysis of optic disk centered (ODC) fundus photographs with a constant image conversion factor (cICF) and individual ICF (iICF) and of macula centered (MC) images with iICF. The y-axis is without units and the exact mean ± standard deviation can be found in the supplement material Table S1. Two-sided paired samples T-test or related samples Wilcoxon signed rank test were used for the comparison of the different methods. The significance threshold was Bonferroni-corrected to < 0.0042.

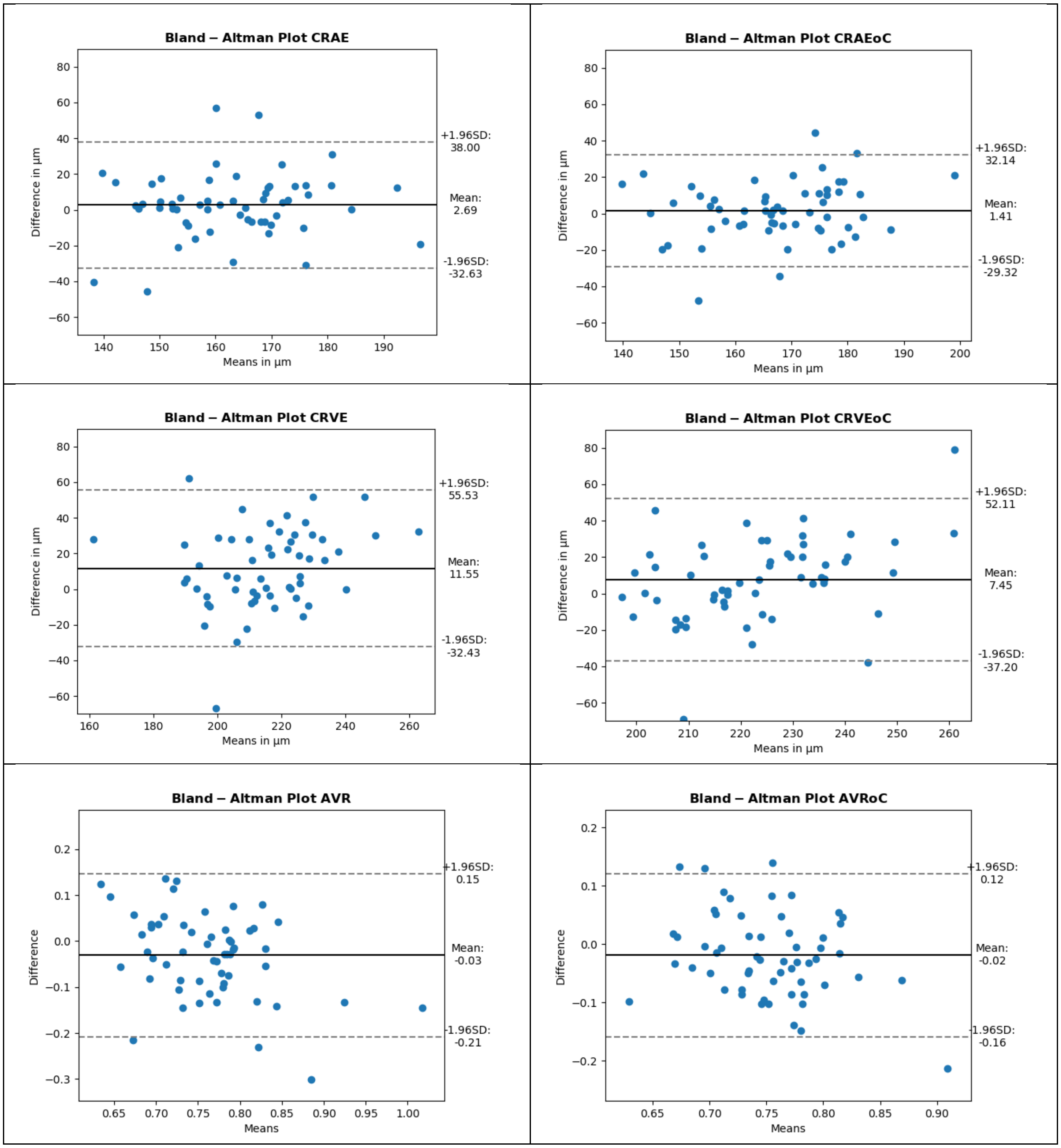

Figure S3:

Bland-Altman plots for optic disk centered (ODC) individual image conversion factor (ICF) versus ODC constant ICF for diameter measurements (central retinal equivalents of arteries and veins (CRAE, CRVE)) and arteriovenous ratio (AVR).

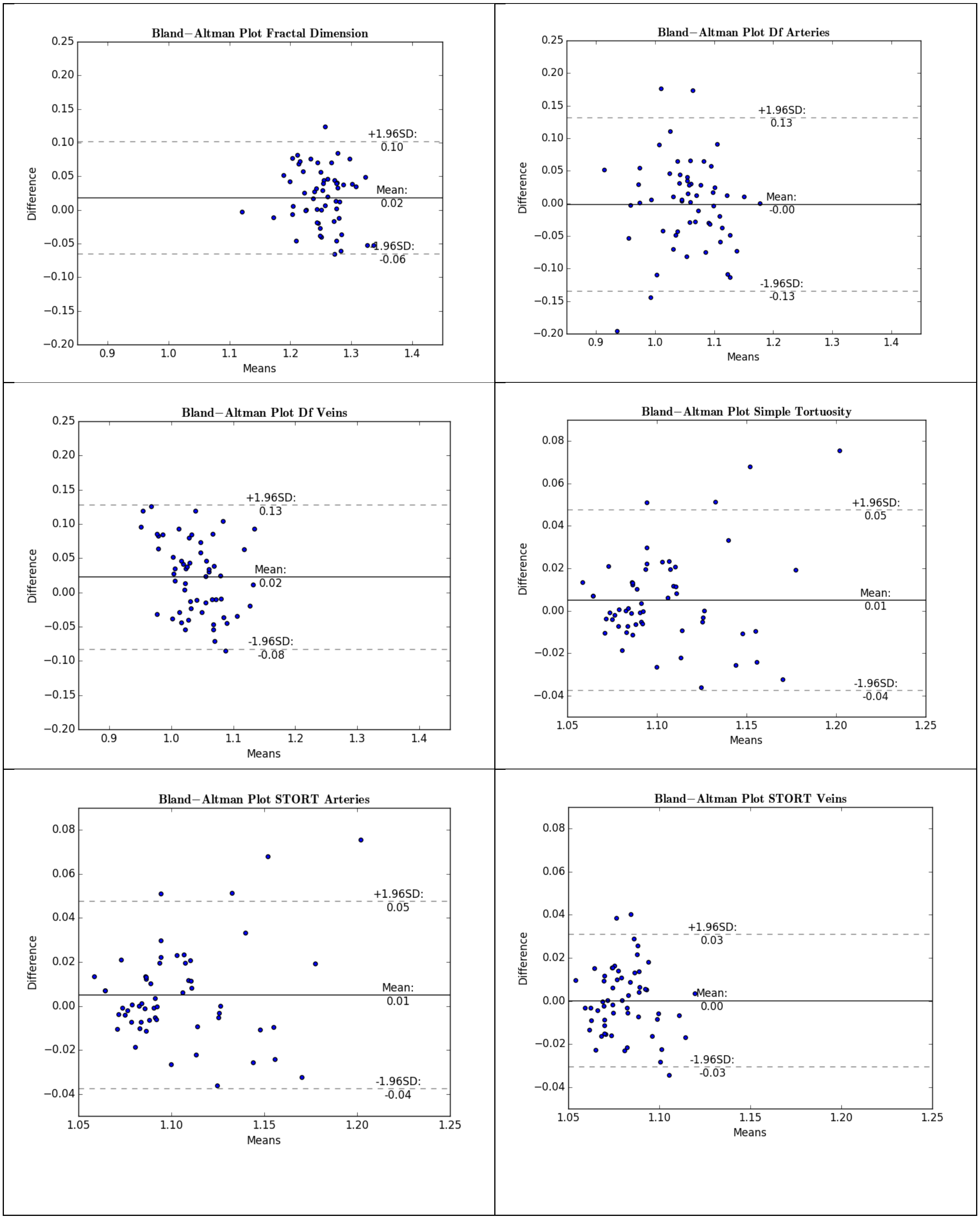

Figure S4:

Bland-Altman plots for optic disk centered (ODC) individual image conversion factor (ICF) versus ODC constant ICF for fractal dimension (Df) and simple tortuosity (STORT) measurements.

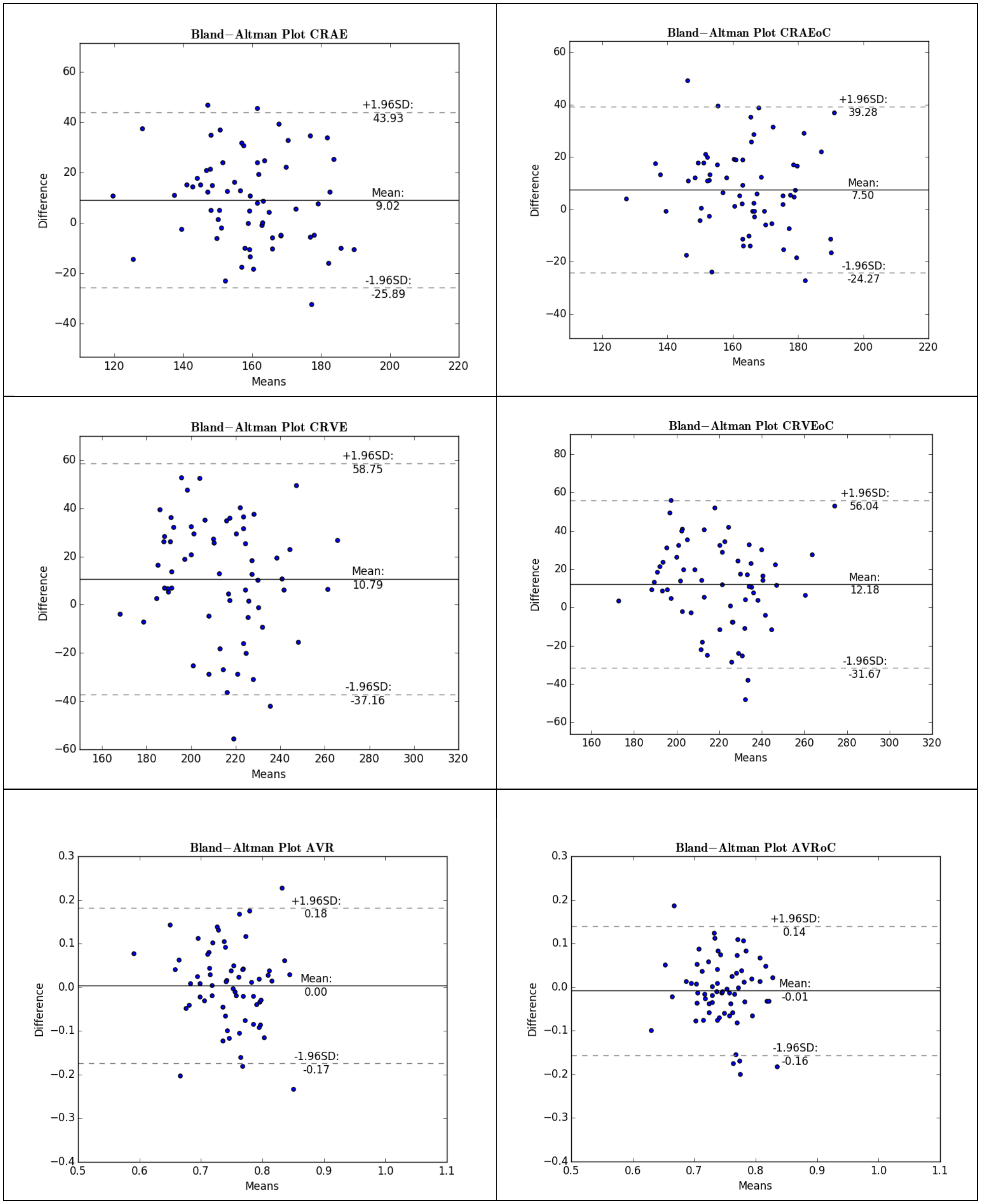

Figure S5:

Bland-Altman analysis for optic disk centered (ODC) individual image conversion factor (ICF) versus macula-centered (MC) individual ICF for diameter measurements (central retinal equivalents of arteries and veins (CRAE, CRVE)) and arteriovenous ratio (AVR).

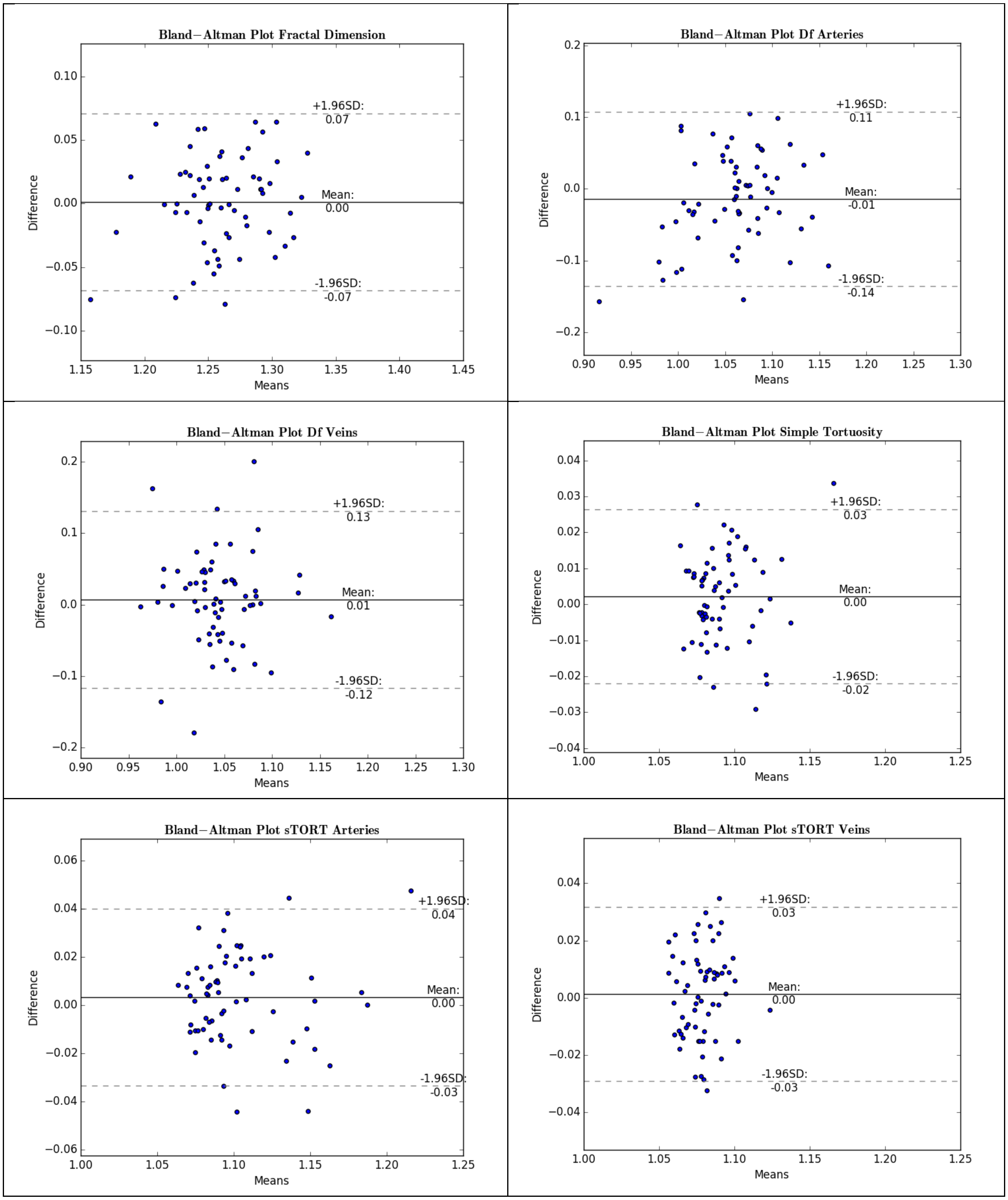

Figure S6:
Bland-Altman analysis for optic disk centered (ODC) individual image conversion factor (ICF) versus macula-centered (MC) individual ICF for fractal dimension (Df) and simple tortuosity (STORT) measurements.

Table S1:
Results of retinal vessel geometric characteristics (RVGC) using two of three analysis methods with SIVA.
The second analysis used ODC images of right eyes with an individual ICF. The third analysis consisted of macula centered images analyzed with an individual ICF. The RVGC are given by their mean ± standard deviation. Two-sided paired samples T-test and related samples Wilcoxon signed rank test (italic values above) results were used for the comparison of the different methods. The significance threshold was Bonferroni-corrected to < 0.0042. * = significant values. CRAEoC = central retinal artery equivalent of zone C, CRVEoC = central retinal vein equivalent of zone C, AVRoC = arteriovenous ratio of zone C, Df = fractal dimension, STt = total simple tortuosity, a = values summarized for arteries, v = values summarized for veins

RVGC	Constant ICF, right eye	Individual ICF	Constant vs. individual ICF, p value (N = 60)	Macula-centered	MC vs. ODC, p value (N = 60)
CRAE, µm	162 ± 14.4	163 ± 15.6	0.269	155 ± 18.4	0.001*
CRVE, µm	208 ± 19.0	219 ± 22.3	*< 0.001**	209 ± 25.1	0.001*
AVR	0.78 ± 0.096	0.75 ± 0.065	*0.027*	0.75 ± 0.074	0.860
CRAEoC, µm	167± 13.2	167 ± 15.2	0.505	160 ± 17.1	0.002*
CRVEoC, µm	218 ± 14.6	225 ± 21.4	*0.007*	215 ± 24.4	*< 0.001**
AVRoC	0.76 ± 0.070	0.74 ± 0.053	0.040	0.75 ± 0.062	0.499
Df	1.25 ± 0.045	1.26 ± 0.040	0.001*	1.26 ± 0.036	*0.800*
Dfa	1.07 ± 0.062	1.05 ± 0.061	0.946	1.07 ± 0.048	*0.093*
Dfv	1.03 ± 0.053	1.05 ± 0.048	0.004*	1.04 ± 0.049	0.301
STt	1.09 ± 0.020	1.09 ± 0.020	*0.497*	1.09 ± 0.020	*0.080*
STa	1.10 ± 0.033	1.10 ± 0.033	*0.270*	1.10 ± 0.034	*0.092*
STv	1.08 ± 0.015	1.08 ± 0.015	*0.711*	1.08 ± 0.014	0.534
